# Investigation of nitro–nitrito photoisomerization: crystal structures of *trans*-bis­(acetyl­acetonato-*O*,*O*′)(pyridine/4-methyl­pyridine/3-hy­droxy­pridine)nitro­cobalt(III)

**DOI:** 10.1107/S2056989018014731

**Published:** 2018-10-23

**Authors:** Shigeru Ohba, Masanobu Tsuchimoto, Hiroki Miyazaki

**Affiliations:** a Research and Education Center for Natural Sciences, Keio University, Hiyoshi 4-1-1 , Kohoku-ku, Yokohama 223-8521, Japan; b Department of Chemistry, Chiba Institute of Technology, Shibazono 2-1-1, Narashino, Chiba 275-0023, Japan; c Department of Chemistry, Faculty of Science and Technology, Keio University, Hiyoshi 3-14-1, Kohoku-ku, Yokohama 223-8522, Japan

**Keywords:** crystal structure, nitro-nitrito photo linkage-isomerization, reaction cavity

## Abstract

The reaction cavities of the nitro groups in the title compounds, *trans*-bis­(acetyl­acetonato-κ^2^ *O*,*O*′)(nitro)(pyridine-κ*N*)cobalt(III), [Co(C_5_H_7_O_2_)_2_(NO_2_)(C_5_H_5_N)], (I), *trans*-bis­(acetyl­acetonato-κ^2^ *O*,*O*′)(4-methyl­pyridine-κ*N*)(nitro)cobalt(III), [Co(C_5_H_7_O_2_)_2_(NO_2_)(C_6_H_7_N)], (II), and *trans*-bis­(acetyl­acetonato-κ^2^ *O*,*O*′)(3-hy­droxy­pyridine-κ*N*)(nitro)cobalt(III) monohydrate, [Co(C_5_H_7_O_2_)_2_(NO_2_)(C_5_H_5_NO)]·H_2_O, (III), have been investigated to reveal that bifurcated inter­molecular C(py)—H⋯O,O contacts in (III) are unfeasible for the nitro–nitrito photochemical linkage isomerization process. In each structure, the pyridine ring and the Co atom lie on a crystallographic mirror plane; in (I) and (II) the nitro group lies in the same plane, whereas in (III), which crystallizes as a monohydrate, the nitro group is disordered over three orientations in a 0.672 (16):0.164 (8):0.164 (8) ratio; the water mol­ecule of crystallization is statistically disordered over two sites adjacent to the mirror plane. In the crystals of (I) and (II), the mol­ecules are linked into [100] chains by C—H⋯O hydrogen bonds, whereas the extended structure of (III) features (010) layers linked by O—H⋯O and C—H⋯O hydrogen bonds. Compounds (I) and (II) were refined as inversion twins.

## Abstract 2

Study of the crystal structures of the title compounds reveals that the solid-state photochemical nitro–nitrito linkage isomerization is restricted by inter­molecular C—H⋯O,O contacts in the 3-hy­droxy­pyridine phase.

## Chemical context   

Solid-state reactions are restricted by the cage effect, which is helpful for stereo-selectivity, but it sometimes inter­rupts the reaction. The photochemical nitro–nitrito linkage isomerization in crystals was investigated for the salts of [Co(NH_3_)_5_(NO_2_)]^+^, and indicated that insufficient free space around the nitro ligand prevents the isomerization from occurring (Boldyreva, 2001 ▸). For the salts of *trans*-[Co(en)_2_(NO_2_)(NCS)]^+^, a certain geometry of the inter­molecular N—H⋯O hydrogen bonds restricts the photoisomerization (Ohba *et al.*, 2018 ▸). In the present study, we investigated another type of nitro­cobalt complex, *trans*-[Co(acac)_2_(NO_2_)(*X*-py)], where acac stands for acetyl­acetonate ion, and *X*-py = pyridine (I) or pyridine derivative; 4-Me-py (II), 3-OH-py (III), and 3-Me-py (IV). The photoactivity of (I) in the solid state had been reported based on the infrared spectra while irradiated with a high-pressure mercury arc, a remarkable increase in absorption in the region 1000–1050 cm^−1^ being detected (Johnson & Martin, 1969 ▸). This is due to the symmetric N—O stretching mode of the nitrito form, and it corresponds to 1055 cm^−1^ for [Co(NH_3_)_5_ONO]Cl_2_ (Heyns & de Waal, 1989 ▸).
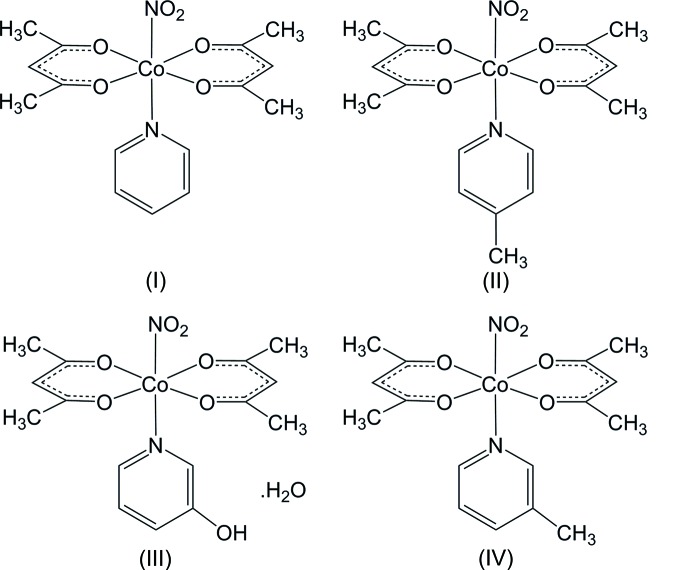


When the IR spectra were measured after irradiation for 30 min to the KBr disks containing each complex by a 150 W Xe lamp without filtering, those of py (I) and 4-Me-py (II) showed an apparent increase of an absorption peak at 1051 and 1025 cm^−1^, respectively (see the figure in the supporting information), and the spectra reverted to those before irradiation on standing at room temperature for *ca* 16 h. The changing color of the KBr disks by photoirradiation was ambiguous, which might be due to the dark-red color of the crystals. On the other hand, the 3-OH-py (III) and 3-Me-py (IV) complexes were photo-stable and did not show the change in IR spectra by irradiation. In the present study, the crystal structures of (I)–(III) have been determined to reveal the differences in the circumstances of the nitro ligand. The structure of (IV) was reported previously (Miyazaki *et al.*, 1998 ▸).

## Structural commentary   

**Figure 1:**
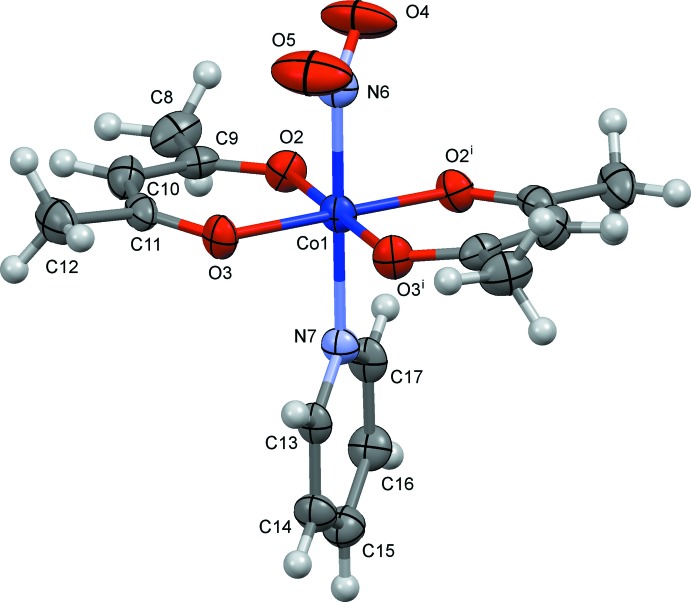
The mol­ecular structure of (I), showing displacement ellipsoids at the 30% probability level. Symmetry code: (i) *x*, −*y* + 1, *z*.

The mol­ecular structures of (I)–(III) are shown in Figs. 1 ▸–3 ▸ ▸, respectively. In these crystals, the complex has crystallographic mirror symmetry, and the py/4-Me-py/3-OH-py ligands and the cobalt atom lie on a mirror plane. The nitro group also lies on the mirror plane in (I) and (II). However, in (III) the nitro group shows positional disorder, and the major component [O4—N8—O4^i^, 67.2 (16)%] is oriented perpendicular to the mirror plane. The minor component [O5*A*—N8—O5*B*, 16.4 (8)%] and the water mol­ecule (O7) are disordered near the mirror. The Co—N(nitro) bond distances are 1.923 (9) Å in (I), 1.949 (10) Å in (II) and 1.915 (3) Å in (III). In each case, a distorted *trans*-CoN_2_O_4_ octa­hedral coordination polyhedron arises.

## Supra­molecular features   

**Figure 4:**
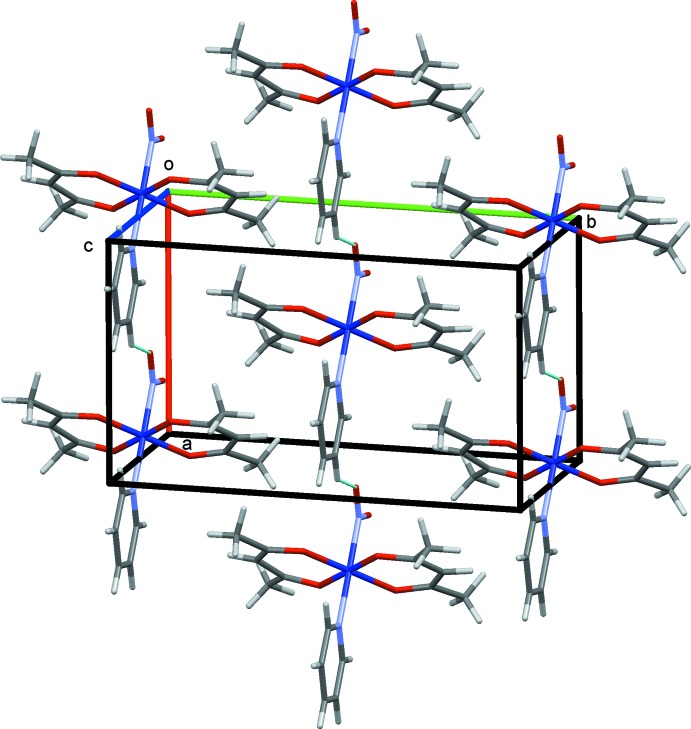
The crystal structure of (I), projected along *c*. The C—H⋯O hydrogen bonds are shown as blue dashed lines.

The crystal structures of (I)–(III) are shown in Figs. 4 ▸–6 ▸ ▸, respectively. In (I) and (II), the mol­ecules are connected by C—H⋯O hydrogen bonds (Tables 1–3 ▸ ▸ ▸), forming chains propagating along the *a-*axis direction. In (III), the complex mol­ecules are connected *via* O—H⋯O hydrogen bonds involving the water mol­ecules, forming layers lying parallel to (010).

**Figure 7:**
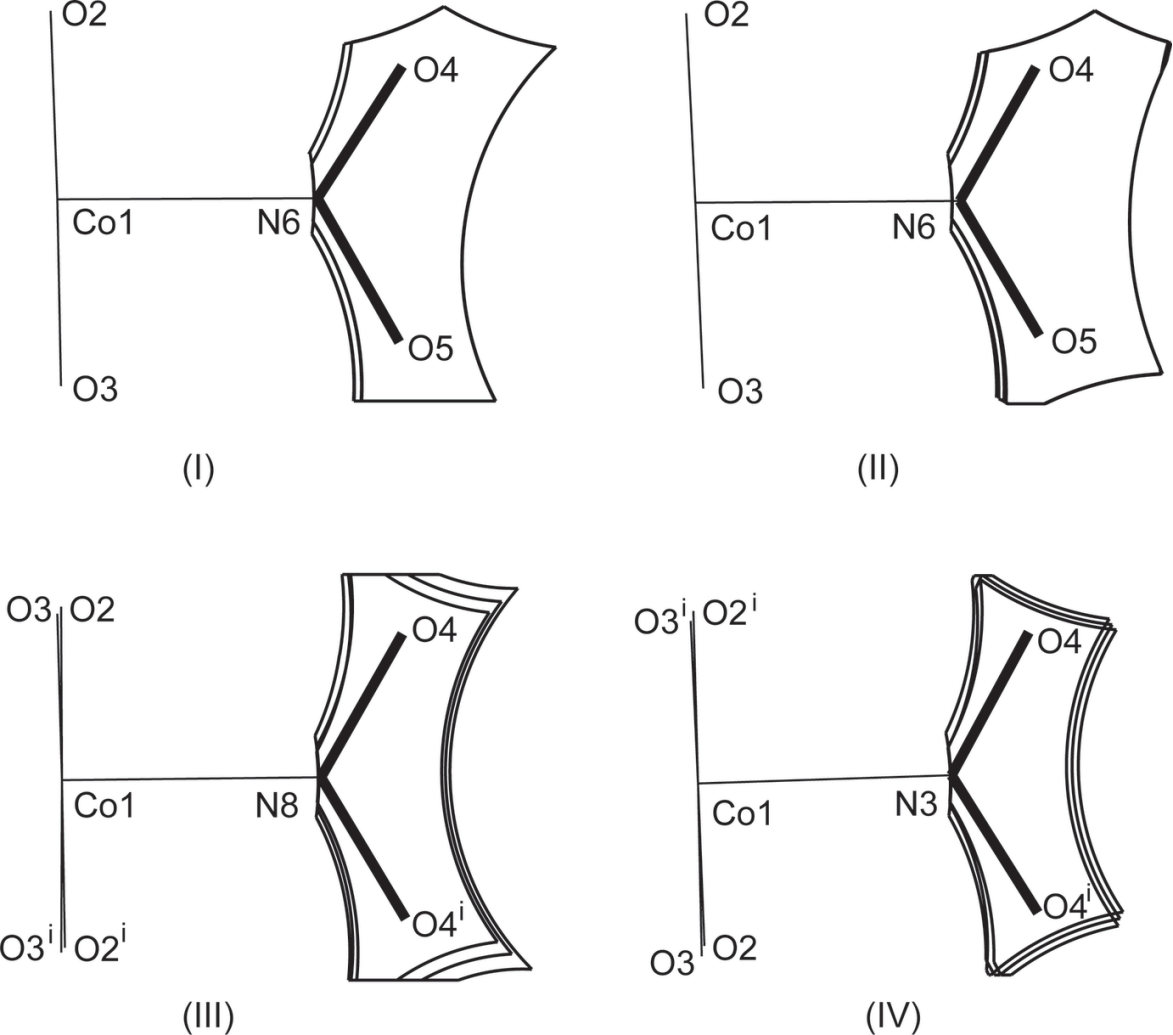
Comparison of the slices of the cavity around the nitro group within 0.1 Å from the plane in (I)–(IV). Symmetry code for (III) (i) *x*, −*y* + 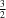, *z*; for (IV): (i) *x*, −*y* + 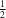, *z*.

**Figure 8:**
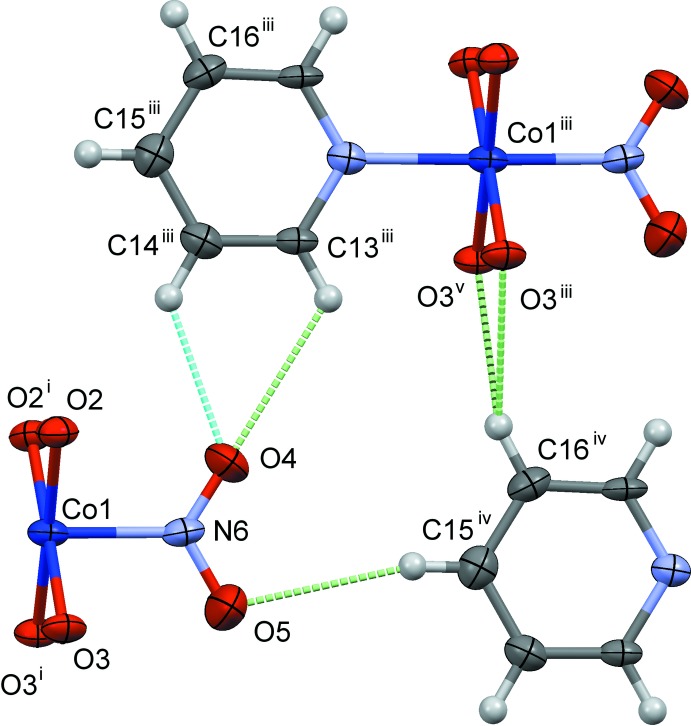
The steric circumstance of the nitro group in (I). Only parts of the complex are shown for clarity. The C—H⋯O hydrogen bonds are shown as blue dashed lines. The green dashed lines indicate other O⋯H contacts shorter than 2.8 Å, O5⋯H15^iv^=2.73 Å. Symmetry codes: (i) *x*, −*y* + 1, *z*; (ii) *x* + 1, *y*, *z*; (iii) *x* − 1, *y*, *z*; (iv) *x* − 1, *y*, *z* − 1; (v) *x* − 1, −*y* + 1, *z*.

**Figure 10:**
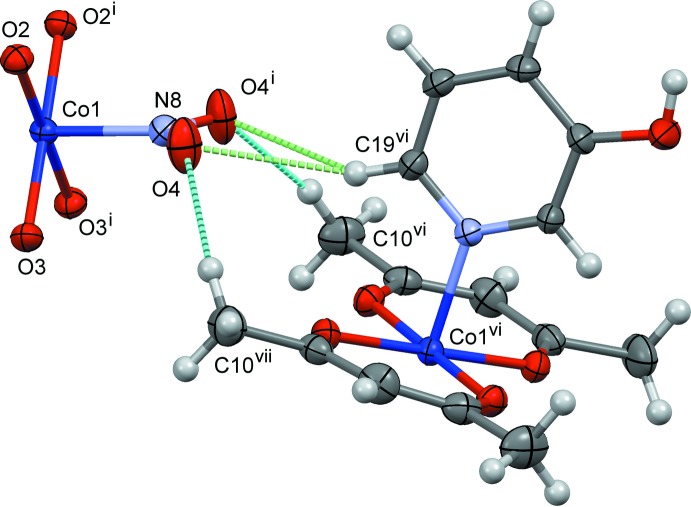
The steric circumstance of the nitro group in (III). Only parts of the complex are shown for clarity. The C—H⋯O hydrogen bonds are shown as blue dashed lines, O4⋯H10*C* ^vii^ = 2.53 Å. The green dashed lines indicate the other O⋯H contacts, O4⋯H19^vi^ = 2.71 Å. Symmetry codes: (i) *x*, −*y* + 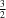, *z*; (ii) *x*, −*y* + 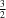, *z* + 1; (iii) *x* + 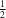, −*y* + 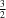, −*z* + 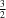; (iv) *x* + 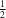, *y*, −*z* + 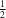; (v) *x* + 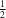, −*y* + 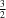, −*z* + 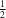; (vi) *x* − 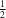, −*y* + 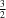, −*z* + 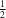; (vii) *x* − 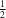, *y*, 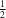 − *z*.

Slices of the reaction cavities around the nitro group near its plane in (I)–(IV) are compared in Fig. 7 ▸, where the radii of the neighboring atoms are assumed to be 1.0 Å greater than the corresponding van der Waals radii (Bondi, 1964 ▸) except for Co, its radius being set to 1.90 Å. The inter­molecular contacts that define the shape of cavity of NO_2_^−^ in its place in (I)–(IV) are shown in Figs. 8 ▸–11 ▸ ▸ ▸, respectively, where the C—H⋯O hydrogen bonds are shown as blue dashed lines (the O⋯H distances being in the range from 2.39 to 2.53 Å), and other O⋯H contacts of less than 2.8 Å are shown as green dashed lines. The cavities in the photo-stable crystals of (III) and (IV) are thinner than those in the photo-active ones (I) and (II), where it seems that there are no close contacts that prevents the linkage isomerization (Figs. 8 ▸ and 9 ▸). The narrow cavities in (III) and (IV) are due to the bifurcated inter­molecular C—H(py)⋯O,O(nitro) contacts as seen in Figs. 10 ▸ and 11 ▸. On the extension of the Co–N(nitro) bond axis, there is a neighboring pyridine ring perpendicular to the nitro plane, suggesting that this ring will block the rotation of NO_2_^−^ to become a nitrito form.

## Database survey   

There are two entries of *trans*-[Co(acac)_2_(NO_2_)(*X*-py)] in the Cambridge Structural Database (CSD Version 5.39; Groom *et al.*, 2016 ▸), the pyridine derivative being 3-methyl­pyridine (Miyazaki *et al.*, 1998 ▸), and 4,4,5,5-tetra­methyl-2-(3-pyrid­yl)imidazolin-1-oxyl radical (Ogita *et al.*, 2002 ▸). Entries for the other related compounds include *trans*-[Co(acac)_2_(NO_2_)(2-amino­pyrimidine)] (Kistenmacher *et al.*, 1978 ▸), *trans*-[Co(acac)_2_(NO_2_)(H_2_O)] (Englert & Strähle, 1987 ▸), and *trans*-[Co(acac)_2_(4-methylpyridine)_2_]PF_6_ (Tayyari *et al.*, 2015 ▸), for which theoretical assignments of the IR bands were presented.

## Synthesis and crystallization   

The title compounds were prepared according to the method of Boucher & Bailar (1965 ▸) from Na[Co(acac)_2_(NO_2_)_2_] and the appropriate pyridine derivative. Dark-red plates of (I), dark-red prisms of (II) and dark-red needles of (III) were grown from aceto­nitrile, nitro­methane and methanol solutions, respectively.

## Refinement   

Crystal data, data collection and structure refinement details are summarized in Table 4 ▸. The H atoms bound to C were positioned geometrically, the methyl H atoms being introduced by an HFIX 137 command. They were refined as riding, with C—H = 0.93–0.96 Å, and *U*_iso_(H) = 1.2*U*_eq_(C) or 1.5*U*_eq_(C_meth­yl_). (I): two reflections showing poor agreement with *I*_obs_ much smaller than *I*_calc_ were omitted from the final refinement. (II): one reflection showing poor agreement was omitted. The DELU instruction was applied to C15 and C18 to avoid the 10 s.u. of the Hirshfeld test difference. (III): six reflections showing poor agreement were omitted. The minor occupancy nitro atoms O5*A* and O5*B* were refined anisotropically with an ISOR instruction. The H atoms bound to O were positioned from difference density maps, and their positional parameters were refined with the geometry restrained and with *U*_iso_(H) = 1.5*U*_eq_(O). Compounds (I) and (II) were refined as inversion twins.

## Supplementary Material

Crystal structure: contains datablock(s) I, II, III, general. DOI: 10.1107/S2056989018014731/hb7778sup1.cif

Structure factors: contains datablock(s) I. DOI: 10.1107/S2056989018014731/hb7778Isup2.hkl

Supporting information file. DOI: 10.1107/S2056989018014731/hb7778Isup5.cdx

Structure factors: contains datablock(s) II. DOI: 10.1107/S2056989018014731/hb7778IIsup3.hkl

Supporting information file. DOI: 10.1107/S2056989018014731/hb7778IIsup6.cdx

Structure factors: contains datablock(s) III. DOI: 10.1107/S2056989018014731/hb7778IIIsup4.hkl

Supporting information file. DOI: 10.1107/S2056989018014731/hb7778IIIsup8.cdx

The IR spectra of py (I) and 4-Me-py (II) compounds before and after photoirradiation for 30 min by a 150 W Xe lamp to the KBr disks.. DOI: 10.1107/S2056989018014731/hb7778sup9.tif

CCDC references: 1873929, 1873928, 1873927

Additional supporting information:  crystallographic information; 3D view; checkCIF report

## Figures and Tables

**Figure 2:**
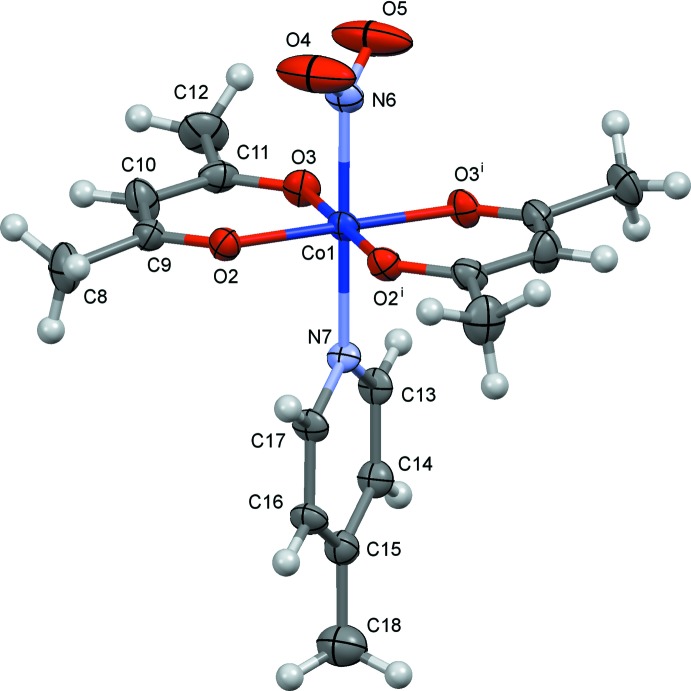
The mol­ecular structure of (II), showing displacement ellipsoids at the 30% probability level. Symmetry code: (i) *x*, −*y* + 1, *z*. One of the two set of H-atom positions of the C18 methyl group is omitted for clarity.

**Figure 3:**
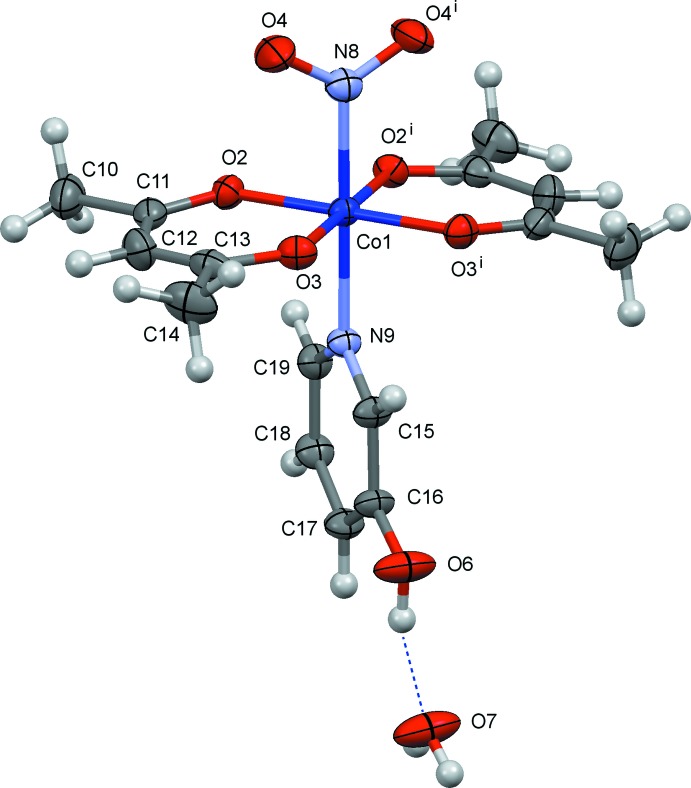
The mol­ecular structure of (III), showing displacement ellipsoids at the 30% probability level. Symmetry code: (i) *x*, −*y* + 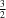, *z*. The minor occupancy O5*A*/O5*B* atoms of the nitro group and one of two possible positions of the water mol­ecule O7 are omitted for clarity.

**Figure 5:**
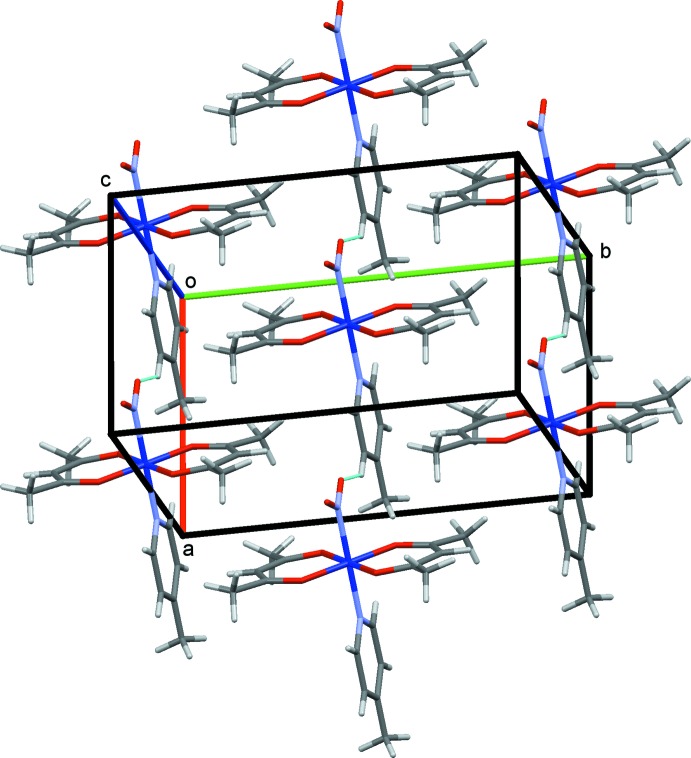
The crystal structure of (II), projected along *c*. The C—H⋯O hydrogen bonds are shown as blue dashed lines.

**Figure 6:**
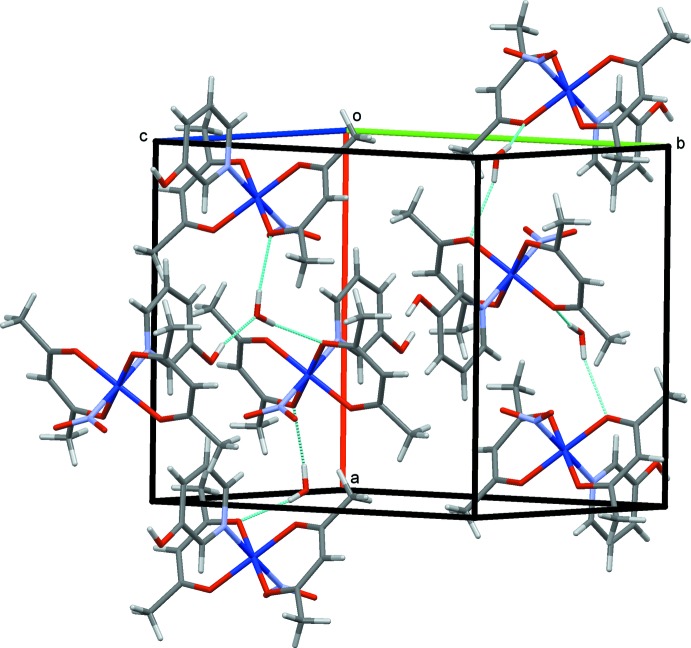
The crystal structure of (III), projected along *c*. The O—H⋯O hydrogen bonds are shown as blue dashed lines. The minor occupancy O5*A*/O5*B* atoms of the nitro group and one of two possible positions of the water mol­ecule O7 are omitted for clarity.

**Figure 9:**
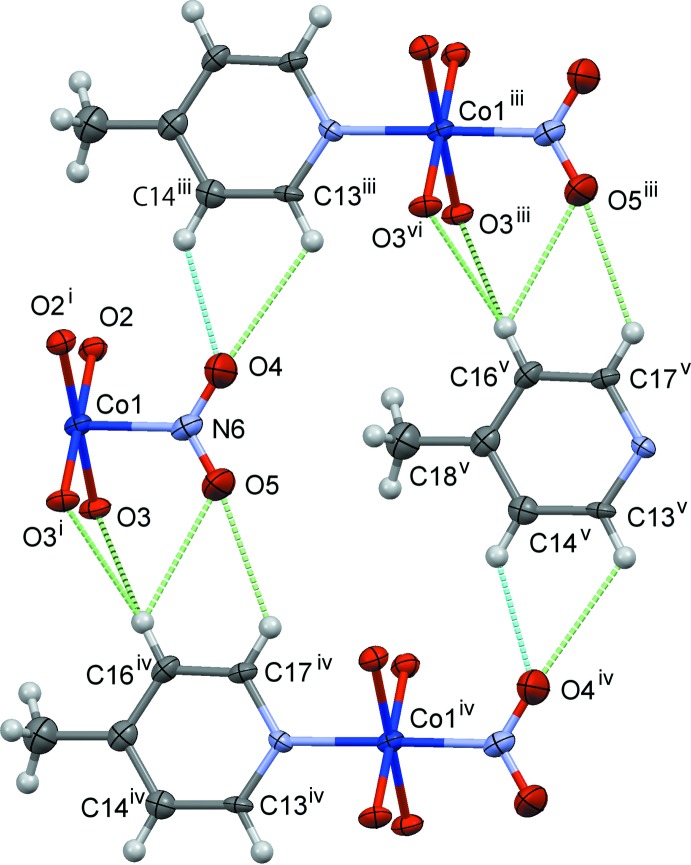
The steric circumstance of the nitro group in (II). Only parts of the complex are shown for clarity. The C—H⋯O hydrogen bonds are shown as blue dashed lines. The green dashed lines indicate other O⋯H contacts shorter than 2.8 Å. Symmetry codes: (i) *x*, −*y* + 1, *z*; (ii) *x* + 1, *y*, *z*; (iii) *x* − 1, *y*, *z*; (iv) *x*, *y*, *z* + 1; (v) *x* − 1, *y*, *z* + 1; (vi) *x* − 1, −*y* + 1, *z*.

**Figure 11:**
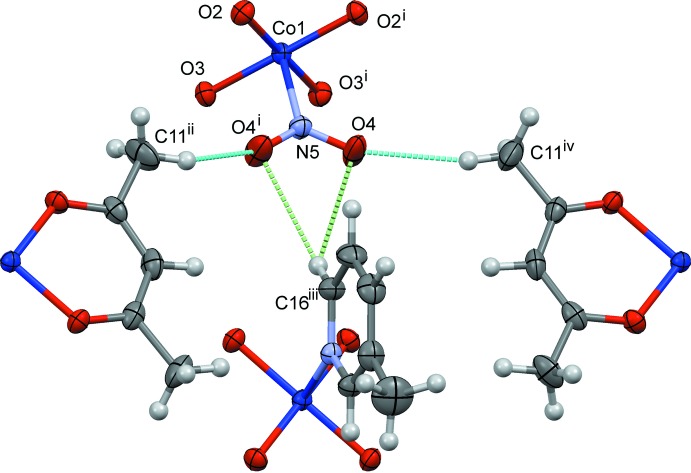
The steric circumstance of the nitro group in (IV). Only parts of the complexes are shown for clarity. The C—H⋯O hydrogen bonds are shown as blue dashed lines, O4⋯H11*A* ^iv^ = 2.41 Å. The green dashed lines indicate the other O⋯H contacts, O4⋯H16^iii^ = 2.69 Å. Symmetry codes: (i) *x*, −*y* + 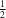, *z*; (ii) −*x* + 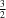, −*y*, *z* + 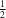; (iii) *x* + 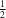, *y*, −*z* + 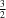; (iv) −*x* + 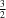, *y* − 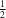, *z* + 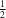.

**Table 1:** Hydrogen-bond geometry (Å, °) for (I)

*D*—H⋯*A*	*D*—H	H⋯*A*	*D*⋯*A*	*D*—H⋯*A*
C14—H14⋯O4^ii^	0.93	2.47	3.150 (11)	130

Symmetry code: (ii) 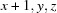.

**Table 2:** Hydrogen-bond geometry (Å, °) for (II)

*D*—H⋯*A*	*D*—H	H⋯*A*	*D*⋯*A*	*D*—H⋯*A*
C14—H14⋯O4^ii^	0.93	2.39	3.104 (10)	133

Symmetry code: (ii) 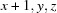.

**Table 3:** Hydrogen-bond geometry (Å, °) for (III)

*D*—H⋯*A*	*D*—H	H⋯*A*	*D*⋯*A*	*D*—H⋯*A*
O6—H6⋯O7	0.84 (2)	1.77 (2)	2.593 (4)	166 (3)
O6—H6⋯O7^i^	0.84 (2)	1.77 (2)	2.593 (4)	166 (3)
O7—H7*A*⋯O2^ii^	0.83 (2)	2.15 (3)	2.962 (4)	165 (8)
O7—H7*B*⋯O3^iii^	0.83 (2)	2.23 (3)	3.030 (5)	164 (8)
C10—H10*C*⋯O4^iv^	0.96	2.53	3.446 (5)	161
C19—H19⋯O5*A* ^iv^	0.93	2.49	3.413 (11)	171
C19—H19⋯O5*A* ^v^	0.93	2.49	3.413 (11)	171

Symmetry codes: (i) 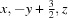; (ii) 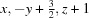; (iii) 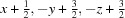; (iv) 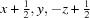; (v) 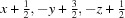.

**Table 4:** Experimental details

	(I)	(II)	(III)
Crystal data			
Chemical formula	[Co(C_5_H_7_O_2_)_2_(NO_2_)(C_5_H_5_N)]	[Co(C_5_H_7_O_2_)_2_(NO_2_)(C_6_H_7_N)]	[Co(C_5_H_7_O_2_)_2_(NO_2_)(C_5_H_5_NO)]·H_2_O
*M*_r_	382.25	396.28	416.27
Crystal system, space group	Monoclinic, *C* *m*	Monoclinic, *C* *m*	Orthorhombic, *P* *n* *m* *a*
Temperature (K)	301	301	301
*a*, *b*, *c* (Å)	8.1971 (14), 13.942 (2), 7.4148 (11)	8.2459 (9), 13.9603 (14), 7.9222 (8)	12.3811 (4), 14.0483 (5), 10.6443 (3)
α, β, γ (°)	90, 91.588 (6), 90	90, 96.997 (4), 90	90, 90, 90
*V* (Å^3^)	847.1 (2)	905.17 (16)	1851.40 (10)
*Z*	2	2	4
Radiation type	Mo *K*α	Mo *K*α	Mo *K*α
μ (mm^−1^)	1.05	0.98	0.97
Crystal size (mm)	0.31 × 0.27 × 0.13	0.35 × 0.15 × 0.15	0.35 × 0.11 × 0.08

Data collection			
Diffractometer	Bruker D8 VENTURE	Bruker D8 VENTURE	Bruker D8 VENTURE
Absorption correction	Integration (*SADABS*; Bruker, 2016 ▸)	Integration (*SADABS*; Bruker, 2016 ▸)	Integration (*SADABS*; Bruker, 2016 ▸)
*T*_min_, *T*_max_	0.731, 0.886	0.749, 0.895	0.780, 0.938
No. of measured, independent and observed [*I* > 2σ(*I*)] reflections	3958, 1529, 1449	4495, 1810, 1754	19560, 2292, 1887
*R*_int_	0.024	0.021	0.032
(sin θ/λ)_max_ (Å^−1^)	0.659	0.660	0.660

Refinement			
*R*[*F* ^2^ > 2σ(*F* ^2^)], *wR*(*F* ^2^), *S*	0.040, 0.083, 1.12	0.031, 0.074, 1.13	0.032, 0.087, 1.10
No. of reflections	1529	1810	2292
No. of parameters	128	134	165
No. of restraints	2	3	16
H-atom treatment	H-atom parameters constrained	H-atom parameters constrained	H atoms treated by a mixture of independent and constrained refinement
Δρ_max_, Δρ_min_ (e Å^−3^)	0.32, −0.34	0.35, −0.37	0.46, −0.46
Absolute structure	Refined as an inversion twin	Refined as an inversion twin	–
Absolute structure parameter	0.41 (3)	0.37 (3)	–

Computer programs: *APEX3* and *SAINT* (Bruker, 2016 ▸), *SHELXT* (Sheldrick, 2015*a* ▸), *Mercury* (Macrae *et al.*, 2008 ▸), *CAVITY* (Ohashi *et al.*, 1981 ▸), *SHELXL2014* (Sheldrick, 2015*b* ▸) and *publCIF* (Westrip, 2010 ▸).

## supplementary crystallographic information

### trans-Bis(acetylacetonato-κ2O,O')(nitro)(pyridine-κN)cobalt(III) (I) Crystal data

**Table:**

[Co(C_5_H_7_O_2_)_2_(NO_2_)(C_5_H_5_N)]	*F*(000) = 396
*M**_r_* = 382.25	*D*_x_ = 1.499 Mg m^−^^3^
Monoclinic, *C**m*	Mo *K*α radiation, λ = 0.71073 Å
*a* = 8.1971 (14) Å	Cell parameters from 2813 reflections
*b* = 13.942 (2) Å	θ = 2.8–27.4°
*c* = 7.4148 (11) Å	µ = 1.05 mm^−^^1^
β = 91.588 (6)°	*T* = 301 K
*V* = 847.1 (2) Å^3^	Plate, dark red
*Z* = 2	0.31 × 0.27 × 0.13 mm

### trans-Bis(acetylacetonato-κ2O,O')(nitro)(pyridine-κN)cobalt(III) (I) Data collection

**Table:**

Bruker D8 VENTURE diffractometer	1449 reflections with *I* > 2σ(*I*)
φ and ω scans	*R*_int_ = 0.024
Absorption correction: integration (SADABS; Bruker, 2016)	θ_max_ = 27.9°, θ_min_ = 2.8°
*T*_min_ = 0.731, *T*_max_ = 0.886	*h* = −8→10
3958 measured reflections	*k* = −17→18
1529 independent reflections	*l* = −8→9

### trans-Bis(acetylacetonato-κ2O,O')(nitro)(pyridine-κN)cobalt(III) (I) Refinement

**Table:**

Refinement on *F*^2^	H-atom parameters constrained
Least-squares matrix: full	*w* = 1/[σ^2^(*F*_o_^2^) + (0.0237*P*)^2^ + 1.1288*P*] where *P* = (*F*_o_^2^ + 2*F*_c_^2^)/3
*R*[*F*^2^ > 2σ(*F*^2^)] = 0.040	(Δ/σ)_max_ = 0.013
*wR*(*F*^2^) = 0.083	Δρ_max_ = 0.32 e Å^−^^3^
*S* = 1.12	Δρ_min_ = −0.34 e Å^−^^3^
1529 reflections	Extinction correction: SHELXL2014 (Sheldrick, 2015b), Fc^*^=kFc[1+0.001xFc^2^λ^3^/sin(2θ)]^-1/4^
128 parameters	Extinction coefficient: 0.0076 (16)
2 restraints	Absolute structure: Refined as an inversion twin
Hydrogen site location: inferred from neighbouring sites	Absolute structure parameter: 0.41 (3)

### trans-Bis(acetylacetonato-κ2O,O')(nitro)(pyridine-κN)cobalt(III) (I) Special details

**Table:**

Geometry. All esds (except the esd in the dihedral angle between two l.s. planes) are estimated using the full covariance matrix. The cell esds are taken into account individually in the estimation of esds in distances, angles and torsion angles; correlations between esds in cell parameters are only used when they are defined by crystal symmetry. An approximate (isotropic) treatment of cell esds is used for estimating esds involving l.s. planes.
Refinement. Refined as a 2-component inversion twin.

### trans-Bis(acetylacetonato-κ2O,O')(nitro)(pyridine-κN)cobalt(III) (I) Fractional atomic coordinates and isotropic or equivalent isotropic displacement parameters (Å^2^)

**Table:**

	*x*	*y*	*z*	*U*_iso_*/*U*_eq_	
Co1	0.42186 (18)	0.5000	0.42933 (17)	0.0438 (3)	
O2	0.3213 (6)	0.5899 (5)	0.5783 (6)	0.0519 (15)	
O3	0.5237 (6)	0.5919 (5)	0.2843 (6)	0.0510 (15)	
O4	0.1030 (10)	0.5000	0.3107 (12)	0.109 (4)	
O5	0.2595 (11)	0.5000	0.1013 (12)	0.124 (4)	
N6	0.2370 (11)	0.5000	0.2638 (11)	0.052 (2)	
N7	0.6127 (10)	0.5000	0.6056 (11)	0.046 (2)	
C8	0.2472 (10)	0.7342 (6)	0.7050 (8)	0.068 (2)	
H8A	0.1347	0.7153	0.7085	0.103*	
H8B	0.2537	0.8018	0.6813	0.103*	
H8C	0.3003	0.7203	0.8191	0.103*	
C9	0.3290 (9)	0.6804 (7)	0.5596 (9)	0.050 (2)	
C10	0.4051 (12)	0.7261 (3)	0.4179 (11)	0.0578 (14)	
H10	0.3946	0.7924	0.4090	0.069*	
C11	0.4946 (9)	0.6800 (7)	0.2902 (9)	0.0476 (19)	
C12	0.5655 (11)	0.7380 (7)	0.1361 (9)	0.072 (2)	
H12A	0.6120	0.6952	0.0502	0.107*	
H12B	0.6485	0.7803	0.1834	0.107*	
H12C	0.4803	0.7750	0.0780	0.107*	
C13	0.7701 (12)	0.5000	0.5474 (12)	0.0441 (18)	
H13	0.7884	0.5000	0.4242	0.053*	
C14	0.8993 (9)	0.5000	0.6648 (10)	0.0560 (17)	
H14	1.0050	0.5000	0.6224	0.067*	
C15	0.8733 (10)	0.5000	0.8481 (10)	0.0607 (18)	
H15	0.9607	0.5000	0.9308	0.073*	
C16	0.7142 (10)	0.5000	0.9055 (9)	0.0577 (18)	
H16	0.6931	0.5000	1.0281	0.069*	
C17	0.5893 (11)	0.5000	0.7818 (11)	0.049 (2)	
H17	0.4829	0.5000	0.8222	0.059*	

### trans-Bis(acetylacetonato-κ2O,O')(nitro)(pyridine-κN)cobalt(III) (I) Atomic displacement parameters (Å^2^)

**Table:**

	*U*^11^	*U*^22^	*U*^33^	*U*^12^	*U*^13^	*U*^23^
Co1	0.0433 (4)	0.0486 (4)	0.0404 (4)	0.000	0.0172 (3)	0.000
O2	0.054 (4)	0.058 (4)	0.044 (3)	0.006 (3)	0.017 (3)	−0.001 (3)
O3	0.056 (4)	0.052 (4)	0.046 (3)	−0.004 (3)	0.021 (3)	0.008 (3)
O4	0.040 (4)	0.212 (10)	0.076 (5)	0.000	0.012 (4)	0.000
O5	0.075 (6)	0.242 (12)	0.056 (5)	0.000	−0.006 (4)	0.000
N6	0.055 (6)	0.066 (6)	0.035 (4)	0.000	0.017 (4)	0.000
N7	0.043 (5)	0.051 (5)	0.045 (4)	0.000	0.010 (3)	0.000
C8	0.083 (5)	0.072 (5)	0.050 (4)	0.021 (4)	−0.008 (3)	−0.020 (4)
C9	0.040 (4)	0.062 (5)	0.047 (4)	0.009 (3)	−0.006 (3)	−0.004 (3)
C10	0.071 (4)	0.048 (2)	0.054 (3)	−0.002 (4)	−0.002 (3)	0.005 (4)
C11	0.051 (4)	0.051 (4)	0.040 (3)	−0.013 (3)	−0.005 (3)	0.013 (3)
C12	0.070 (5)	0.083 (6)	0.062 (4)	−0.020 (4)	0.007 (4)	0.019 (4)
C13	0.048 (5)	0.044 (4)	0.041 (4)	0.000	0.021 (3)	0.000
C14	0.044 (4)	0.064 (4)	0.061 (4)	0.000	0.012 (3)	0.000
C15	0.065 (5)	0.061 (4)	0.055 (4)	0.000	−0.004 (4)	0.000
C16	0.070 (5)	0.068 (5)	0.035 (3)	0.000	0.009 (3)	0.000
C17	0.055 (5)	0.056 (4)	0.039 (4)	0.000	0.029 (4)	0.000

### trans-Bis(acetylacetonato-κ2O,O')(nitro)(pyridine-κN)cobalt(III) (I) Geometric parameters (Å, º)

**Table:**

Co1—O2^i^	1.877 (5)	C9—C10	1.392 (11)
Co1—O2	1.877 (5)	C10—C11	1.374 (12)
Co1—O3	1.883 (6)	C10—H10	0.9300
Co1—O3^i^	1.883 (6)	C11—C12	1.527 (9)
Co1—N6	1.923 (9)	C12—H12A	0.9600
Co1—N7	2.010 (8)	C12—H12B	0.9600
O2—C9	1.270 (10)	C12—H12C	0.9600
O3—C11	1.252 (10)	C13—C14	1.352 (12)
O4—N6	1.162 (11)	C13—H13	0.9300
O5—N6	1.224 (12)	C14—C15	1.381 (10)
N7—C17	1.325 (11)	C14—H14	0.9300
N7—C13	1.372 (12)	C15—C16	1.383 (10)
C8—C9	1.489 (10)	C15—H15	0.9300
C8—H8A	0.9600	C16—C17	1.356 (12)
C8—H8B	0.9600	C16—H16	0.9300
C8—H8C	0.9600	C17—H17	0.9300
			
O2^i^—Co1—O2	83.8 (4)	O2—C9—C8	113.4 (8)
O2^i^—Co1—O3	178.7 (3)	C10—C9—C8	122.4 (9)
O2—Co1—O3	95.18 (10)	C11—C10—C9	124.4 (4)
O2^i^—Co1—O3^i^	95.18 (10)	C11—C10—H10	117.8
O2—Co1—O3^i^	178.7 (3)	C9—C10—H10	117.8
O3—Co1—O3^i^	85.8 (4)	O3—C11—C10	126.1 (7)
O2^i^—Co1—N6	91.4 (3)	O3—C11—C12	114.6 (8)
O2—Co1—N6	91.4 (3)	C10—C11—C12	119.3 (8)
O3—Co1—N6	89.4 (2)	C11—C12—H12A	109.5
O3^i^—Co1—N6	89.4 (2)	C11—C12—H12B	109.5
O2^i^—Co1—N7	87.9 (2)	H12A—C12—H12B	109.5
O2—Co1—N7	87.9 (2)	C11—C12—H12C	109.5
O3—Co1—N7	91.3 (3)	H12A—C12—H12C	109.5
O3^i^—Co1—N7	91.3 (3)	H12B—C12—H12C	109.5
N6—Co1—N7	179.1 (5)	C14—C13—N7	121.6 (8)
C9—O2—Co1	125.1 (6)	C14—C13—H13	119.2
C11—O3—Co1	124.1 (6)	N7—C13—H13	119.2
O4—N6—O5	117.7 (10)	C13—C14—C15	119.6 (7)
O4—N6—Co1	122.9 (8)	C13—C14—H14	120.2
O5—N6—Co1	119.4 (8)	C15—C14—H14	120.2
C17—N7—C13	118.2 (8)	C16—C15—C14	118.4 (7)
C17—N7—Co1	120.7 (7)	C16—C15—H15	120.8
C13—N7—Co1	121.1 (7)	C14—C15—H15	120.8
C9—C8—H8A	109.5	C17—C16—C15	119.5 (6)
C9—C8—H8B	109.5	C17—C16—H16	120.2
H8A—C8—H8B	109.5	C15—C16—H16	120.2
C9—C8—H8C	109.5	N7—C17—C16	122.7 (8)
H8A—C8—H8C	109.5	N7—C17—H17	118.7
H8B—C8—H8C	109.5	C16—C17—H17	118.7
O2—C9—C10	124.2 (8)		
			
O2^i^—Co1—O2—C9	−178.3 (4)	Co1—O3—C11—C10	10.4 (11)
O3—Co1—O2—C9	2.5 (6)	Co1—O3—C11—C12	−168.5 (5)
N6—Co1—O2—C9	−87.0 (6)	C9—C10—C11—O3	−1.6 (15)
N7—Co1—O2—C9	93.6 (6)	C9—C10—C11—C12	177.2 (7)
O2—Co1—O3—C11	−9.7 (6)	C17—N7—C13—C14	0.000 (2)
O3^i^—Co1—O3—C11	171.1 (4)	Co1—N7—C13—C14	180.000 (1)
N6—Co1—O3—C11	81.7 (6)	N7—C13—C14—C15	0.000 (2)
N7—Co1—O3—C11	−97.7 (6)	C13—C14—C15—C16	0.000 (2)
Co1—O2—C9—C10	4.4 (10)	C14—C15—C16—C17	0.000 (2)
Co1—O2—C9—C8	−175.9 (4)	C13—N7—C17—C16	0.000 (2)
O2—C9—C10—C11	−6.6 (14)	Co1—N7—C17—C16	180.000 (2)
C8—C9—C10—C11	173.8 (7)	C15—C16—C17—N7	0.000 (3)

Symmetry code: (i) *x*, −*y*+1, *z*.

### trans-Bis(acetylacetonato-κ2O,O')(nitro)(pyridine-κN)cobalt(III) (I) Hydrogen-bond geometry (Å, º)

**Table:**

*D*—H···*A*	*D*—H	H···*A*	*D*···*A*	*D*—H···*A*
C14—H14···O4^ii^	0.93	2.47	3.150 (11)	130

Symmetry code: (ii) *x*+1, *y*, *z*.

### trans-Bis(acetylacetonato-κ2O,O')(4-methylpyridine-κN)(nitro)cobalt(III) (II) Crystal data

**Table:**

[Co(C_5_H_7_O_2_)_2_(NO_2_)(C_6_H_7_N)]	*F*(000) = 412
*M**_r_* = 396.28	*D*_x_ = 1.454 Mg m^−^^3^
Monoclinic, *C**m*	Mo *K*α radiation, λ = 0.71073 Å
*a* = 8.2459 (9) Å	Cell parameters from 4544 reflections
*b* = 13.9603 (14) Å	θ = 2.5–27.8°
*c* = 7.9222 (8) Å	µ = 0.98 mm^−^^1^
β = 96.997 (4)°	*T* = 301 K
*V* = 905.17 (16) Å^3^	Prism, dark red
*Z* = 2	0.35 × 0.15 × 0.15 mm

### trans-Bis(acetylacetonato-κ2O,O')(4-methylpyridine-κN)(nitro)cobalt(III) (II) Data collection

**Table:**

Bruker D8 VENTURE diffractometer	1754 reflections with *I* > 2σ(*I*)
φ and ω scans	*R*_int_ = 0.021
Absorption correction: integration (SADABS; Bruker, 2016)	θ_max_ = 28.0°, θ_min_ = 2.9°
*T*_min_ = 0.749, *T*_max_ = 0.895	*h* = −10→8
4495 measured reflections	*k* = −17→18
1810 independent reflections	*l* = −9→10

### trans-Bis(acetylacetonato-κ2O,O')(4-methylpyridine-κN)(nitro)cobalt(III) (II) Refinement

**Table:**

Refinement on *F*^2^	Hydrogen site location: inferred from neighbouring sites
Least-squares matrix: full	H-atom parameters constrained
*R*[*F*^2^ > 2σ(*F*^2^)] = 0.031	*w* = 1/[σ^2^(*F*_o_^2^) + (0.0113*P*)^2^ + 1.2247*P*] where *P* = (*F*_o_^2^ + 2*F*_c_^2^)/3
*wR*(*F*^2^) = 0.074	(Δ/σ)_max_ = 0.001
*S* = 1.13	Δρ_max_ = 0.35 e Å^−^^3^
1810 reflections	Δρ_min_ = −0.37 e Å^−^^3^
134 parameters	Absolute structure: Refined as an inversion twin
3 restraints	Absolute structure parameter: 0.37 (3)

### trans-Bis(acetylacetonato-κ2O,O')(4-methylpyridine-κN)(nitro)cobalt(III) (II) Special details

**Table:**

Geometry. All esds (except the esd in the dihedral angle between two l.s. planes) are estimated using the full covariance matrix. The cell esds are taken into account individually in the estimation of esds in distances, angles and torsion angles; correlations between esds in cell parameters are only used when they are defined by crystal symmetry. An approximate (isotropic) treatment of cell esds is used for estimating esds involving l.s. planes.
Refinement. Refined as a 2-component inversion twin.

### trans-Bis(acetylacetonato-κ2O,O')(4-methylpyridine-κN)(nitro)cobalt(III) (II) Fractional atomic coordinates and isotropic or equivalent isotropic displacement parameters (Å^2^)

**Table:**

	*x*	*y*	*z*	*U*_iso_*/*U*_eq_	Occ. (<1)
Co1	0.42176 (19)	0.5000	0.52127 (18)	0.03463 (18)	
O2	0.3036 (5)	0.5901 (4)	0.3806 (5)	0.0400 (12)	
O3	0.5407 (5)	0.5913 (4)	0.6616 (5)	0.0439 (13)	
O4	0.1207 (11)	0.5000	0.6291 (11)	0.125 (4)	
O5	0.2965 (10)	0.5000	0.8253 (10)	0.125 (4)	
N6	0.2587 (12)	0.5000	0.6799 (11)	0.048 (2)	
N7	0.5891 (9)	0.5000	0.3642 (10)	0.0330 (18)	
C8	0.2352 (11)	0.7359 (5)	0.2501 (9)	0.065 (3)	
H8A	0.3087	0.7499	0.1683	0.097*	
H8B	0.1957	0.7946	0.2930	0.097*	
H8C	0.1447	0.6991	0.1968	0.097*	
C9	0.3220 (9)	0.6804 (6)	0.3917 (9)	0.043 (2)	
C10	0.4208 (15)	0.7268 (2)	0.5206 (12)	0.0577 (10)	
H10	0.4215	0.7934	0.5206	0.069*	
C11	0.5191 (9)	0.6796 (6)	0.6500 (9)	0.046 (2)	
C12	0.6150 (13)	0.7373 (6)	0.7949 (8)	0.072 (3)	
H12A	0.5936	0.7117	0.9024	0.108*	
H12B	0.5813	0.8032	0.7868	0.108*	
H12C	0.7298	0.7331	0.7857	0.108*	
C13	0.7505 (10)	0.5000	0.4153 (10)	0.0363 (16)	
H13	0.7851	0.5000	0.5315	0.044*	
C14	0.8686 (8)	0.5000	0.3045 (8)	0.0468 (14)	
H14	0.9787	0.5000	0.3475	0.056*	
C15	0.8232 (8)	0.5000	0.1291 (8)	0.0463 (14)	
C16	0.6569 (8)	0.5000	0.0745 (7)	0.0450 (14)	
H16	0.6209	0.5000	−0.0414	0.054*	
C17	0.5439 (10)	0.5000	0.1900 (10)	0.0393 (17)	
H17	0.4333	0.5000	0.1491	0.047*	
C18	0.9477 (16)	0.5000	0.0121 (19)	0.078 (3)	
H18A	0.9079	0.5349	−0.0889	0.117*	0.5
H18B	0.9714	0.4352	−0.0174	0.117*	0.5
H18C	1.0454	0.5299	0.0660	0.117*	0.5

### trans-Bis(acetylacetonato-κ2O,O')(4-methylpyridine-κN)(nitro)cobalt(III) (II) Atomic displacement parameters (Å^2^)

**Table:**

	*U*^11^	*U*^22^	*U*^33^	*U*^12^	*U*^13^	*U*^23^
Co1	0.0384 (4)	0.0391 (3)	0.0248 (3)	0.000	−0.0026 (2)	0.000
O2	0.045 (3)	0.041 (3)	0.033 (3)	0.005 (2)	−0.001 (2)	0.001 (3)
O3	0.047 (3)	0.049 (3)	0.032 (3)	−0.002 (3)	−0.010 (2)	−0.007 (3)
O4	0.059 (5)	0.261 (12)	0.056 (5)	0.000	0.015 (4)	0.000
O5	0.066 (6)	0.271 (12)	0.039 (4)	0.000	0.009 (4)	0.000
N6	0.056 (6)	0.057 (6)	0.029 (5)	0.000	−0.002 (4)	0.000
N7	0.021 (4)	0.044 (4)	0.033 (5)	0.000	−0.001 (3)	0.000
C8	0.072 (6)	0.053 (5)	0.065 (5)	0.009 (4)	−0.015 (5)	0.013 (5)
C9	0.052 (5)	0.044 (5)	0.034 (4)	0.009 (3)	0.005 (4)	0.001 (3)
C10	0.080 (3)	0.0389 (17)	0.051 (2)	−0.007 (6)	−0.0049 (18)	0.005 (6)
C11	0.053 (5)	0.049 (5)	0.037 (4)	−0.008 (3)	0.008 (4)	−0.013 (3)
C12	0.104 (7)	0.068 (5)	0.042 (5)	−0.021 (6)	0.002 (5)	−0.023 (5)
C13	0.026 (3)	0.047 (3)	0.031 (4)	0.000	−0.014 (3)	0.000
C14	0.043 (4)	0.050 (3)	0.047 (4)	0.000	0.002 (3)	0.000
C15	0.055 (4)	0.043 (3)	0.041 (4)	0.000	0.007 (3)	0.000
C16	0.055 (4)	0.055 (3)	0.026 (3)	0.000	0.004 (3)	0.000
C17	0.040 (4)	0.052 (4)	0.024 (3)	0.000	−0.007 (3)	0.000
C18	0.073 (8)	0.098 (5)	0.064 (5)	0.000	0.012 (4)	0.000

### trans-Bis(acetylacetonato-κ2O,O')(4-methylpyridine-κN)(nitro)cobalt(III) (II) Geometric parameters (Å, º)

**Table:**

Co1—O2	1.874 (5)	C10—H10	0.9300
Co1—O2^i^	1.874 (5)	C11—C12	1.539 (9)
Co1—O3	1.886 (5)	C12—H12A	0.9600
Co1—O3^i^	1.886 (5)	C12—H12B	0.9600
Co1—N6	1.949 (10)	C12—H12C	0.9600
Co1—N7	1.968 (8)	C13—C14	1.388 (11)
O2—C9	1.271 (8)	C13—H13	0.9300
O3—C11	1.248 (9)	C14—C15	1.394 (9)
O4—N6	1.160 (12)	C14—H14	0.9300
O5—N6	1.156 (11)	C15—C16	1.387 (9)
N7—C13	1.343 (10)	C15—C18	1.464 (14)
N7—C17	1.385 (10)	C16—C17	1.383 (11)
C8—C9	1.475 (10)	C16—H16	0.9300
C8—H8A	0.9600	C17—H17	0.9300
C8—H8B	0.9600	C18—H18A	0.9600
C8—H8C	0.9600	C18—H18B	0.9600
C9—C10	1.387 (11)	C18—H18C	0.9600
C10—C11	1.392 (11)		
			
O2—Co1—O2^i^	84.4 (3)	C9—C10—H10	118.0
O2—Co1—O3	95.28 (9)	C11—C10—H10	118.0
O2^i^—Co1—O3	179.6 (3)	O3—C11—C10	125.9 (7)
O2—Co1—O3^i^	179.6 (3)	O3—C11—C12	114.0 (7)
O2^i^—Co1—O3^i^	95.29 (9)	C10—C11—C12	120.1 (8)
O3—Co1—O3^i^	85.0 (3)	C11—C12—H12A	109.5
O2—Co1—N6	91.9 (3)	C11—C12—H12B	109.5
O2^i^—Co1—N6	91.9 (3)	H12A—C12—H12B	109.5
O3—Co1—N6	88.3 (2)	C11—C12—H12C	109.5
O3^i^—Co1—N6	88.3 (2)	H12A—C12—H12C	109.5
O2—Co1—N7	88.7 (2)	H12B—C12—H12C	109.5
O2^i^—Co1—N7	88.7 (2)	N7—C13—C14	123.7 (7)
O3—Co1—N7	91.1 (2)	N7—C13—H13	118.1
O3^i^—Co1—N7	91.1 (2)	C14—C13—H13	118.1
N6—Co1—N7	179.1 (5)	C13—C14—C15	120.4 (6)
C9—O2—Co1	125.1 (5)	C13—C14—H14	119.8
C11—O3—Co1	124.4 (5)	C15—C14—H14	119.8
O5—N6—O4	118.7 (11)	C16—C15—C14	116.5 (6)
O5—N6—Co1	121.2 (9)	C16—C15—C18	123.1 (8)
O4—N6—Co1	120.1 (8)	C14—C15—C18	120.5 (8)
C13—N7—C17	115.9 (8)	C17—C16—C15	120.9 (6)
C13—N7—Co1	123.7 (7)	C17—C16—H16	119.5
C17—N7—Co1	120.4 (6)	C15—C16—H16	119.5
C9—C8—H8A	109.5	C16—C17—N7	122.5 (7)
C9—C8—H8B	109.5	C16—C17—H17	118.7
H8A—C8—H8B	109.5	N7—C17—H17	118.7
C9—C8—H8C	109.5	C15—C18—H18A	109.5
H8A—C8—H8C	109.5	C15—C18—H18B	109.5
H8B—C8—H8C	109.5	H18A—C18—H18B	109.5
O2—C9—C10	124.7 (7)	C15—C18—H18C	109.5
O2—C9—C8	115.1 (7)	H18A—C18—H18C	109.5
C10—C9—C8	120.2 (7)	H18B—C18—H18C	109.5
C9—C10—C11	123.9 (3)		
			
O2^i^—Co1—O2—C9	−176.7 (5)	Co1—O3—C11—C12	172.0 (5)
O3—Co1—O2—C9	3.1 (7)	C9—C10—C11—O3	5.3 (18)
N6—Co1—O2—C9	91.5 (6)	C9—C10—C11—C12	−175.5 (9)
N7—Co1—O2—C9	−87.9 (6)	C17—N7—C13—C14	0.000 (1)
O2—Co1—O3—C11	4.5 (7)	Co1—N7—C13—C14	180.000 (1)
O3^i^—Co1—O3—C11	−175.7 (5)	N7—C13—C14—C15	0.000 (1)
N6—Co1—O3—C11	−87.3 (6)	C13—C14—C15—C16	0.000 (1)
N7—Co1—O3—C11	93.3 (6)	C13—C14—C15—C18	180.000 (1)
Co1—O2—C9—C10	−6.9 (12)	C14—C15—C16—C17	0.000 (1)
Co1—O2—C9—C8	170.3 (5)	C18—C15—C16—C17	180.000 (1)
O2—C9—C10—C11	3.4 (18)	C15—C16—C17—N7	0.000 (1)
C8—C9—C10—C11	−173.6 (8)	C13—N7—C17—C16	0.000 (1)
Co1—O3—C11—C10	−8.8 (12)	Co1—N7—C17—C16	180.000 (1)

Symmetry code: (i) *x*, −*y*+1, *z*.

### trans-Bis(acetylacetonato-κ2O,O')(4-methylpyridine-κN)(nitro)cobalt(III) (II) Hydrogen-bond geometry (Å, º)

**Table:**

*D*—H···*A*	*D*—H	H···*A*	*D*···*A*	*D*—H···*A*
C14—H14···O4^ii^	0.93	2.39	3.104 (10)	133

Symmetry code: (ii) *x*+1, *y*, *z*.

### trans-Bis(acetylacetonato-κ2O,O')(3-hydroxypyridine-κN)(nitro)cobalt(III) monohydrate (III) Crystal data

**Table:**

[Co(C_5_H_7_O_2_)_2_(NO_2_)(C_5_H_5_NO)]·H_2_O	*D*_x_ = 1.493 Mg m^−^^3^
*M**_r_* = 416.27	Mo *K*α radiation, λ = 0.71073 Å
Orthorhombic, *P**n**m**a*	Cell parameters from 9558 reflections
*a* = 12.3811 (4) Å	θ = 2.4–27.9°
*b* = 14.0483 (5) Å	µ = 0.97 mm^−^^1^
*c* = 10.6443 (3) Å	*T* = 301 K
*V* = 1851.40 (10) Å^3^	Needle, dark red
*Z* = 4	0.35 × 0.11 × 0.08 mm
*F*(000) = 864	

### trans-Bis(acetylacetonato-κ2O,O')(3-hydroxypyridine-κN)(nitro)cobalt(III) monohydrate (III) Data collection

**Table:**

Bruker D8 VENTURE diffractometer	1887 reflections with *I* > 2σ(*I*)
φ and ω scans	*R*_int_ = 0.032
Absorption correction: integration (SADABS; Bruker, 2016)	θ_max_ = 28.0°, θ_min_ = 2.5°
*T*_min_ = 0.780, *T*_max_ = 0.938	*h* = −16→15
19560 measured reflections	*k* = −18→17
2292 independent reflections	*l* = −14→14

### trans-Bis(acetylacetonato-κ2O,O')(3-hydroxypyridine-κN)(nitro)cobalt(III) monohydrate (III) Refinement

**Table:**

Refinement on *F*^2^	16 restraints
Least-squares matrix: full	Hydrogen site location: mixed
*R*[*F*^2^ > 2σ(*F*^2^)] = 0.032	H atoms treated by a mixture of independent and constrained refinement
*wR*(*F*^2^) = 0.087	*w* = 1/[σ^2^(*F*_o_^2^) + (0.0204*P*)^2^ + 2.0145*P*] where *P* = (*F*_o_^2^ + 2*F*_c_^2^)/3
*S* = 1.10	(Δ/σ)_max_ < 0.001
2292 reflections	Δρ_max_ = 0.46 e Å^−^^3^
165 parameters	Δρ_min_ = −0.46 e Å^−^^3^

### trans-Bis(acetylacetonato-κ2O,O')(3-hydroxypyridine-κN)(nitro)cobalt(III) monohydrate (III) Special details

**Table:**

Geometry. All esds (except the esd in the dihedral angle between two l.s. planes) are estimated using the full covariance matrix. The cell esds are taken into account individually in the estimation of esds in distances, angles and torsion angles; correlations between esds in cell parameters are only used when they are defined by crystal symmetry. An approximate (isotropic) treatment of cell esds is used for estimating esds involving l.s. planes.

### trans-Bis(acetylacetonato-κ2O,O')(3-hydroxypyridine-κN)(nitro)cobalt(III) monohydrate (III) Fractional atomic coordinates and isotropic or equivalent isotropic displacement parameters (Å^2^)

**Table:**

	*x*	*y*	*z*	*U*_iso_*/*U*_eq_	Occ. (<1)
Co1	0.35185 (3)	0.7500	0.39108 (4)	0.03386 (13)	
O2	0.44528 (12)	0.66083 (11)	0.31791 (14)	0.0410 (3)	
O3	0.25897 (12)	0.66025 (12)	0.46516 (14)	0.0439 (4)	
O4	0.2376 (4)	0.6750 (2)	0.1940 (3)	0.076 (3)	0.672 (16)
O5A	0.1771 (13)	0.7215 (16)	0.2462 (12)	0.078 (12)	0.164 (8)
O5B	0.3040 (13)	0.7736 (15)	0.1426 (10)	0.076 (13)	0.164 (8)
O6	0.4018 (2)	0.7500	0.8767 (2)	0.0756 (10)	
H6	0.446 (3)	0.7500	0.936 (4)	0.113*	
O7	0.5265 (3)	0.7776 (6)	1.0691 (3)	0.088 (4)	0.5
H7A	0.502 (5)	0.784 (6)	1.141 (3)	0.132*	0.5
H7B	0.5927 (18)	0.785 (6)	1.068 (6)	0.132*	0.5
N8	0.2659 (2)	0.7500	0.2415 (3)	0.0440 (6)	
N9	0.44358 (19)	0.7500	0.5455 (2)	0.0359 (5)	
C10	0.5278 (2)	0.5157 (2)	0.2729 (3)	0.0669 (8)	
H10A	0.5146	0.5128	0.1841	0.100*	
H10B	0.5295	0.4523	0.3067	0.100*	
H10C	0.5960	0.5462	0.2880	0.100*	
C11	0.43951 (19)	0.57141 (17)	0.3353 (2)	0.0454 (5)	
C12	0.3602 (2)	0.52607 (18)	0.4046 (3)	0.0581 (7)	
H12	0.3649	0.4603	0.4123	0.070*	
C13	0.2749 (2)	0.57064 (18)	0.4630 (2)	0.0493 (6)	
C14	0.1918 (3)	0.5134 (2)	0.5334 (3)	0.0771 (9)	
H14A	0.2089	0.5138	0.6214	0.116*	
H14B	0.1920	0.4491	0.5030	0.116*	
H14C	0.1217	0.5409	0.5208	0.116*	
C15	0.3987 (2)	0.7500	0.6592 (3)	0.0416 (7)	
H15	0.3237	0.7500	0.6647	0.050*	
C16	0.4571 (3)	0.7500	0.7686 (3)	0.0440 (7)	
C17	0.5695 (3)	0.7500	0.7601 (3)	0.0449 (7)	
H17	0.6122	0.7500	0.8320	0.054*	
C18	0.6153 (3)	0.7500	0.6424 (3)	0.0449 (7)	
H18	0.6901	0.7500	0.6343	0.054*	
C19	0.5519 (2)	0.7500	0.5370 (3)	0.0404 (7)	
H19	0.5843	0.7500	0.4581	0.048*	

### trans-Bis(acetylacetonato-κ2O,O')(3-hydroxypyridine-κN)(nitro)cobalt(III) monohydrate (III) Atomic displacement parameters (Å^2^)

**Table:**

	*U*^11^	*U*^22^	*U*^33^	*U*^12^	*U*^13^	*U*^23^
Co1	0.0317 (2)	0.0381 (2)	0.0318 (2)	0.000	0.00176 (15)	0.000
O2	0.0397 (8)	0.0418 (8)	0.0415 (8)	0.0026 (7)	0.0031 (6)	−0.0036 (7)
O3	0.0383 (8)	0.0528 (9)	0.0407 (8)	−0.0074 (7)	0.0028 (7)	0.0044 (7)
O4	0.106 (6)	0.0559 (19)	0.066 (4)	−0.003 (2)	−0.039 (4)	−0.0118 (16)
O5A	0.053 (7)	0.15 (3)	0.036 (5)	−0.045 (13)	−0.016 (5)	0.009 (9)
O5B	0.069 (8)	0.12 (4)	0.035 (5)	−0.040 (15)	0.009 (5)	0.023 (9)
O6	0.0423 (14)	0.152 (3)	0.0329 (13)	0.000	−0.0008 (11)	0.000
O7	0.0508 (19)	0.175 (12)	0.0375 (16)	−0.014 (3)	−0.0008 (14)	−0.015 (3)
N8	0.0380 (14)	0.0506 (16)	0.0434 (15)	0.000	−0.0011 (12)	0.000
N9	0.0311 (12)	0.0414 (13)	0.0352 (13)	0.000	−0.0007 (10)	0.000
C10	0.0668 (18)	0.0595 (16)	0.0743 (19)	0.0186 (14)	−0.0060 (15)	−0.0180 (15)
C11	0.0503 (13)	0.0424 (12)	0.0435 (12)	0.0061 (10)	−0.0125 (10)	−0.0060 (10)
C12	0.0725 (18)	0.0372 (12)	0.0645 (16)	−0.0016 (12)	−0.0020 (14)	0.0066 (12)
C13	0.0523 (14)	0.0518 (14)	0.0437 (12)	−0.0124 (11)	−0.0083 (11)	0.0127 (11)
C14	0.078 (2)	0.079 (2)	0.075 (2)	−0.0267 (17)	0.0003 (17)	0.0309 (17)
C15	0.0322 (14)	0.0586 (19)	0.0339 (15)	0.000	0.0036 (12)	0.000
C16	0.0418 (17)	0.060 (2)	0.0299 (15)	0.000	0.0006 (13)	0.000
C17	0.0380 (16)	0.0575 (19)	0.0391 (16)	0.000	−0.0096 (13)	0.000
C18	0.0296 (14)	0.0544 (19)	0.0507 (19)	0.000	−0.0025 (13)	0.000
C19	0.0332 (15)	0.0466 (17)	0.0414 (16)	0.000	0.0032 (13)	0.000

### trans-Bis(acetylacetonato-κ2O,O')(3-hydroxypyridine-κN)(nitro)cobalt(III) monohydrate (III) Geometric parameters (Å, º)

**Table:**

Co1—O2^i^	1.8745 (15)	C10—H10A	0.9600
Co1—O2	1.8745 (15)	C10—H10B	0.9600
Co1—O3	1.8799 (15)	C10—H10C	0.9600
Co1—O3^i^	1.8800 (15)	C11—C12	1.384 (4)
Co1—N8	1.915 (3)	C12—C13	1.376 (4)
Co1—N9	1.998 (2)	C12—H12	0.9300
O2—C11	1.272 (3)	C13—C14	1.505 (3)
O3—C13	1.274 (3)	C14—H14A	0.9600
O4—N8	1.220 (3)	C14—H14B	0.9600
O5A—N8	1.172 (13)	C14—H14C	0.9600
O5B—N8	1.200 (13)	C15—C16	1.371 (4)
O6—C16	1.340 (4)	C15—H15	0.9300
O6—H6	0.84 (2)	C16—C17	1.394 (4)
O7—H7A	0.83 (2)	C17—C18	1.376 (5)
O7—H7B	0.83 (2)	C17—H17	0.9300
N8—O4^i^	1.220 (3)	C18—C19	1.370 (4)
N9—C15	1.332 (4)	C18—H18	0.9300
N9—C19	1.344 (4)	C19—H19	0.9300
C10—C11	1.500 (3)		
			
O2^i^—Co1—O2	83.87 (9)	H10A—C10—H10C	109.5
O2^i^—Co1—O3	179.59 (7)	H10B—C10—H10C	109.5
O2—Co1—O3	95.94 (7)	O2—C11—C12	124.9 (2)
O2^i^—Co1—O3^i^	95.94 (7)	O2—C11—C10	114.2 (2)
O2—Co1—O3^i^	179.59 (7)	C12—C11—C10	120.9 (2)
O3—Co1—O3^i^	84.24 (10)	C13—C12—C11	125.2 (2)
O2^i^—Co1—N8	89.85 (8)	C13—C12—H12	117.4
O2—Co1—N8	89.85 (8)	C11—C12—H12	117.4
O3—Co1—N8	90.51 (8)	O3—C13—C12	125.2 (2)
O3^i^—Co1—N8	90.51 (8)	O3—C13—C14	114.4 (3)
O2^i^—Co1—N9	89.49 (7)	C12—C13—C14	120.4 (3)
O2—Co1—N9	89.49 (7)	C13—C14—H14A	109.5
O3—Co1—N9	90.14 (7)	C13—C14—H14B	109.5
O3^i^—Co1—N9	90.14 (7)	H14A—C14—H14B	109.5
N8—Co1—N9	179.12 (11)	C13—C14—H14C	109.5
C11—O2—Co1	124.40 (16)	H14A—C14—H14C	109.5
C13—O3—Co1	124.08 (16)	H14B—C14—H14C	109.5
C16—O6—H6	108 (4)	N9—C15—C16	123.4 (3)
H7A—O7—H7B	111 (5)	N9—C15—H15	118.3
O5A—N8—O5B	120.1 (9)	C16—C15—H15	118.3
O4—N8—O4^i^	119.5 (4)	O6—C16—C15	117.4 (3)
O5A—N8—Co1	119.1 (6)	O6—C16—C17	124.4 (3)
O5B—N8—Co1	120.7 (7)	C15—C16—C17	118.2 (3)
O4—N8—Co1	120.26 (19)	C18—C17—C16	118.1 (3)
O4^i^—N8—Co1	120.26 (19)	C18—C17—H17	121.0
C15—N9—C19	118.6 (3)	C16—C17—H17	121.0
C15—N9—Co1	120.7 (2)	C19—C18—C17	120.6 (3)
C19—N9—Co1	120.7 (2)	C19—C18—H18	119.7
C11—C10—H10A	109.5	C17—C18—H18	119.7
C11—C10—H10B	109.5	N9—C19—C18	121.1 (3)
H10A—C10—H10B	109.5	N9—C19—H19	119.5
C11—C10—H10C	109.5	C18—C19—H19	119.5
			
O2^i^—Co1—O2—C11	173.79 (14)	Co1—O3—C13—C14	177.53 (16)
O3—Co1—O2—C11	−5.84 (18)	C11—C12—C13—O3	−2.6 (4)
N8—Co1—O2—C11	−96.34 (18)	C11—C12—C13—C14	178.5 (2)
N9—Co1—O2—C11	84.25 (18)	C19—N9—C15—C16	0.000 (1)
O2—Co1—O3—C13	4.64 (19)	Co1—N9—C15—C16	180.000 (1)
O3^i^—Co1—O3—C13	−174.99 (15)	N9—C15—C16—O6	180.000 (1)
N8—Co1—O3—C13	94.55 (19)	N9—C15—C16—C17	0.000 (1)
N9—Co1—O3—C13	−84.86 (18)	O6—C16—C17—C18	180.000 (1)
Co1—O2—C11—C12	3.9 (3)	C15—C16—C17—C18	0.000 (1)
Co1—O2—C11—C10	−176.26 (16)	C16—C17—C18—C19	0.000 (1)
O2—C11—C12—C13	1.3 (4)	C15—N9—C19—C18	0.000 (1)
C10—C11—C12—C13	−178.6 (2)	Co1—N9—C19—C18	180.000 (1)
Co1—O3—C13—C12	−1.4 (3)	C17—C18—C19—N9	0.000 (1)

Symmetry code: (i) *x*, −*y*+3/2, *z*.

### trans-Bis(acetylacetonato-κ2O,O')(3-hydroxypyridine-κN)(nitro)cobalt(III) monohydrate (III) Hydrogen-bond geometry (Å, º)

**Table:**

*D*—H···*A*	*D*—H	H···*A*	*D*···*A*	*D*—H···*A*
O6—H6···O7	0.84 (2)	1.77 (2)	2.593 (4)	166 (3)
O6—H6···O7^i^	0.84 (2)	1.77 (2)	2.593 (4)	166 (3)
O7—H7*A*···O2^ii^	0.83 (2)	2.15 (3)	2.962 (4)	165 (8)
O7—H7*B*···O3^iii^	0.83 (2)	2.23 (3)	3.030 (5)	164 (8)
C10—H10*C*···O4^iv^	0.96	2.53	3.446 (5)	161
C19—H19···O5*A*^iv^	0.93	2.49	3.413 (11)	171
C19—H19···O5*A*^v^	0.93	2.49	3.413 (11)	171

Symmetry codes: (i) *x*, −*y*+3/2, *z*; (ii) *x*, −*y*+3/2, *z*+1; (iii) *x*+1/2, −*y*+3/2, −*z*+3/2; (iv) *x*+1/2, *y*, −*z*+1/2; (v) *x*+1/2, −*y*+3/2, −*z*+1/2.
