# Supplementary figures and images for: Investigation of nitro–nitrito photoisomerization: crystal structures of trans-bis­(acetyl­acetonato-O,O′)(pyridine/4-methyl­pyridine/3-hy­droxy­pridine)nitro­cobalt(III)
Source: Acta Crystallogr E Crystallogr Commun. 2018 Oct 23;74(Pt 11):1637–42. doi: 10.1107/S2056989018014731 (PMC6218901; doi:10.1107/S2056989018014731)

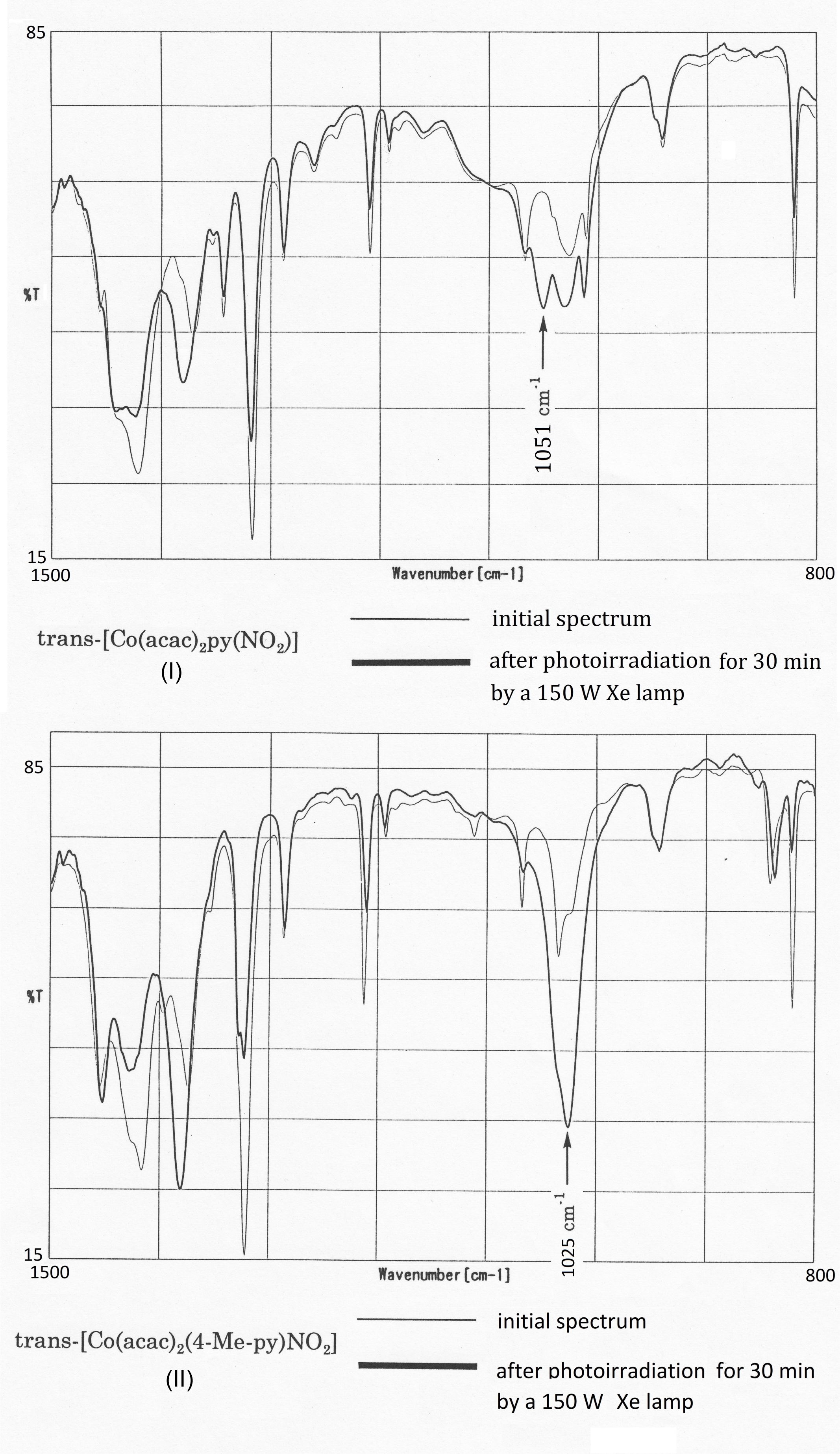

Supplement: Supplementary file 8 [file e-74-01637-sup9.tif]
